# The Gut-Joint Axis in Osteoarthritis: A Bibliometric Analysis of Research Trends and Mechanistic Insights (2008–2025)

**DOI:** 10.2174/0118715303399881251121113531

**Published:** 2026-03-03

**Authors:** Xiaolong Liu, Yuanzhi Zhang, Nan Ye

**Affiliations:** 1 Department of Joint Surgery, the Second Affiliated Hospital of Inner Mongolia Medical University, No.59 South Horqin Road, Saihan District, Hohhot 010010Inner Mongolia, China

**Keywords:** Gut microbiota, osteoarthritis, bibliometric analysis, systemic inflammation, therapeutic strategies, microbial imbalance

## Abstract

**Introduction:**

Osteoarthritis (OA) is a leading cause of disability, and emerging evidence highlights the gut–joint axis as critical in its pathogenesis and therapy. To date, no bibliometric synthesis has comprehensively mapped this field.

**Methods:**

We searched the Web of Science Core Collection (2008–2025) for original studies on gut microbiota and OA. Bibliometric and network analyses were performed using bibliometrix (R) and VOSviewer to assess publication trends, citations, collaborations, and research themes.

**Results:**

A total of 175 original articles were identified, with a marked rise in research activity after 2015 and an accelerated growth post-2021. China and the United States are the leading contributors, with China exhibiting the most extensive international collaborations. The University of Calgary and Central South University emerged as top institutions, while journals, such as *Osteoarthritis and Cartilage*, demonstrated high impact. Thematic analysis revealed seven major research clusters, emphasizing systemic inflammation, metabolic risk factors, molecular mechanisms, and therapeutic strategies targeting the gut-joint axis. Recent years have seen a shift toward translational and mechanistic studies, with increased focus on molecular markers, clinical trials, and precision interventions.

**Discussion:**

The results demonstrate a transition from descriptive and preclinical studies to mechanistic and clinical investigations. Collaborative networks across North America, Europe, and Asia have accelerated progress, but research remains uneven across regions.

**Conclusion:**

This bibliometric analysis underscores the rapid expansion of gut microbiota–OA research, highlighting microbial imbalance as a promising therapeutic target. Future work should integrate multi-omics, clinical trials, and global collaboration to advance precision strategies for OA management.

## 1 INTRODUCTION

Osteoarthritis (OA) is a prevalent age-related degenerative joint disease and one of the leading causes of disability worldwide. Its incidence has been increasing, particularly among individuals over 45 years old, rising from 29.2 per 1,000 person-years in 1992 to 40.5 per 1,000 person-years by 2013. Recognized by the World Health Organization (WHO) as a priority disease [1], OA primarily affects weight-bearing joints, such as the knee and hip, leading to chronic pain, mobility impairment, and reduced quality of life [2]. Current treatments, including pain management, physical therapy, and surgical interventions, are largely palliative, focusing on symptom relief rather than disease modification [3, 4]. As OA prevalence rises, there is an urgent need to elucidate its pathophysiology and develop novel therapeutic strategies.

A growing body of evidence highlights the “gut-joint axis”, the bidirectional relationship between gut microbiota and joint health, as a key factor in OA pathogenesis. The gut microbiota, a diverse microbial community within the gastrointestinal tract, plays a crucial role in maintaining systemic homeostasis and regulating inflammation [5]. Dysbiosis, or imbalance in microbial composition, has been linked to chronic inflammatory diseases, including OA [6, 7]. Gut-derived metabolites, such as lipopolysaccharides (LPS) and short-chain fatty acids (SCFAs), enter the circulation, contributing to low-grade chronic inflammation, a hallmark of OA [8, 9]. Increased intestinal permeability (“leaky gut”) facilitates the translocation of bacterial endotoxins, which can activate inflammatory pathways involving Toll-like receptor 4 (TLR4) and nuclear factor kappa-light-chain-enhancer of activated B cells (NF-κB), leading to the release of cytokines, such as interleukin-6 (IL-6), interleukin-1β (IL-1β), and tumor necrosis factor-alpha (TNF-α). These mediators drive synovial inflammation, cartilage degradation, and subchondral bone remodeling, accelerating OA progression.

In addition to mediating inflammation, gut microbiota also influences metabolic homeostasis and immune responses relevant to OA development. For example, SCFAs, such as butyrate, exert anti-inflammatory effects and regulate immune cells, but dysbiosis is associated with reduced SCFA production and a shift toward a pro-inflammatory state [10-12]. The gut microbiota also affects signaling pathways, including Wnt/β-catenin, MAPK, and JAK-STAT, that are linked to cartilage integrity and chondrocyte survival [13, 14]. Furthermore, oxidative stress and mitochondrial dysfunction, both influenced by microbial alterations, contribute to OA pathophysiology [15].

Given these findings, increasing attention has been paid to microbiota-targeted interventions in OA. Strategies, such as probiotics, prebiotics, fecal microbiota transplantation (FMT), and dietary modification (*e.g*., Mediterranean diet), have been explored for their potential to restore microbial balance, reduce inflammation, and improve joint health in OA [16-20]. As a result, the gut-joint axis and its underlying mechanisms have become a promising multidisciplinary research frontier.

Despite expanding interest, research on the gut-joint axis in OA remains fragmented, employing diverse methodologies and resulting in a complex knowledge landscape. To date, no comprehensive bibliometric mapping or quantitative synthesis has systematically characterized the intellectual structure, research trends, and collaborative networks in this field [21, 22]. Bibliometric analysis, which has proven invaluable in other emerging biomedical disciplines [21, 23-25], offers a robust approach to synthesizing interdisciplinary research and highlighting evolving themes and knowledge gaps.

In this context, our study applies bibliometric analysis to systematically characterize the development, impact, and trajectory of gut microbiota research in osteoarthritis over the past two decades. Through this quantitative synthesis, we aim to provide a data-driven overview of current trends, collaborative networks, and emerging thematic directions, thereby informing future research priorities and supporting the advancement of this rapidly expanding field.

## 2 MATERIALS AND METHODS

### 2.1 Search Strategies and Data Collection

To conduct a comprehensive bibliometric analysis of the relationship between gut microbiota and OA, we systematically searched the Web of Science Core Collection (WoSCC), which is widely regarded as a robust source for bibliometric studies [22]. Our search strategy was carefully designed to provide comprehensive coverage and specificity, ensuring the inclusion of relevant literature while maintaining methodological rigor and reproducibility. Drawing upon established bibliometric methodologies and previous studies in this field [21, 22], the search formula was iteratively developed and refined based on terminology trends and expert consensus. The final search string employed was: (TS= (“Gut microbiota” OR “Gut microflora” OR “Gut microbiome” OR “Gut microorganism” OR “gastrointestinal microbiota” OR “gastrointestinal microflora” OR “gastrointestinal microbiome” OR “gastrointestinal microorganism” OR “Intestinal microbiota” OR “Intestinal microflora” OR “Intestinal microbiome” OR “Intestinal microorganism”)) AND (TS=(“Osteoarthritis” OR “osteoarthritides”)). We determined the inclusion and exclusion criteria as follows: Only articles published in English were included to ensure consistency and data quality. In line with bibliometric best practices [21], we excluded review articles, conference abstracts, and non-research communications, retaining only original, full-length research articles to focus on primary research contributions. The search covered articles published from January 2008 to June 2025.

To further enhance the relevance and comprehensiveness of our dataset, we reviewed recent bibliometric analyses and OA-gut axis literature [22, 23]. Based on reviewer feedback, we also cross-referenced our search terms with established gene and microbial species markers associated with OA and gut health (*e.g*., “Akkermansia”, “Prevotella”, “Firmicutes”, “Bacteroidetes”, “SCFA”, “microbial gene”); however, these more granular terms yielded minimal additional unique articles in this bibliometric framework, confirming the adequacy of our primary search strategy.

Following retrieval, all records were exported in “Full Records and References” and “Plain Text” formats, as recommended for compatibility with bibliometric analysis tools. To minimize temporal bias, literature retrieval was performed on a single day (June 23^rd^, 2025), thereby avoiding fluctuations due to database updates.

### 2.2 Statistical Analysis

Bibliometric data were analyzed using a combination of established and innovative tools. Microsoft Excel 16 was used to manage and calculate key indicators, including annual publication volume, citation frequency, average citation rates, journal impact factors (IF), and descriptive statistics on countries, institutions, and authors.

For visual exploration and network mapping, three advanced platforms were employed: VOSviewer (version 1.6.20), CiteSpace (version 6.3 R1), and R4.3.3 VOSviewer supported the construction and visualization of institutional and author collaboration, co-authorship, and co-citation networks [26]. CiteSpace was used for keyword co-occurrence and burst analysis, with annual time slicing and a node threshold (N=5) to identify evolving trends and research hotspots. Network pruning was conducted using Pathfinder and network-merging techniques, yielding time-sliced visualizations of keyword dynamics. The R4.3.3 (https://bibliometric.com/), a leading online bibliometrics analysis platform [online resource], was also utilized to generate graphical representations of publication trends, geographical distributions, and collaboration networks. This platform is recognized for its intuitive interface and powerful visualization of national, institutional, and author collaboration patterns, keyword analysis, and citation networks.

To quantitatively assess scholarly impact, we calculated the H-index, m-index, and g-index for individual researchers, based on their publication and citation records in WoSCC. Journal impact factors and Journal Citation Reports (JCR) categorizations were used to evaluate journal influence, in accordance with standard bibliometric reporting [21]. The H-index reflects both productivity and citation impact; the m-index adjusts for career length; and the g-index highlights the influence of highly cited papers. These metrics collectively offer a nuanced view of author, institution, and journal impact in the field. Although bibliometric analyses are typically favored for larger datasets, there is precedent for their application in more focused research domains with moderate document counts [21]. This approach allows mapping of intellectual structure, identification of research trends, and detection of collaboration, even in fields with manageable literature volumes, providing value where traditional reviews may miss broader patterns. By restricting our analysis to original articles and leveraging multiple visualization platforms, this study offers both granularity and a comprehensive overview, in line with recent methodological recommendations.

## 3 RESULTS

### 3.1 Publication Trends and Citation Impact in Gut Microbiota and Osteoarthritis Research

The literature selection process is outlined in Fig. (**1**). This study reviewed 175 articles published from 2008 to 2025, authored by 1,228 individuals across 745 institutions in 102 countries/regions (Fig. **2A**). These articles were published in 117 journals and collectively cited 7,700 references. Analysis of publication trends, as shown in Fig. (**2B**), demonstrated a noticeable upward trajectory in research activity on the gut microbiota–osteoarthritis axis, particularly after 2021. From 2012 to 2020, annual publication counts remained low and sporadic, averaging 0–2 per year. A notable increase began in 2021, with 4 publications, followed by a sharp rise to 8 publications in 2022 and a peak of 14 publications in 2023. Although the number declined to 8 publications in 2024, it remained considerably higher than in the preceding decade. In 2025, 10 publications have already been recorded, indicating sustained research interest. The cumulative publication curve (yellow bars) further underscores the field's accelerating growth in recent years. The quadratic regression model applied to these data (y = 0.1884x^2^ – 0.0713x + 0.9059) accounts for 92.1% of the variance (R^2^ = 0.921), underscoring a strong upward trend in research output and reflecting the increasing attention this interdisciplinary topic has received in the scientific community.

“Metformin: Mechanisms in Human Obesity and Weight Loss,” was published in Current Obesity Reports (IF=8.8) in 2019 and has garnered 168 citations [27]. This article is significant for its examination of metabolic treatments and their role in obesity management. The second most cited article, “Intestinal Microbiome Composition and Its Relation to Joint Pain and Inflammation,” was published in Nature Communications (IF=16.6) in 2019 and has accumulated 159 citations [7]. This article highlights the significant relationships between intestinal microbiome profiles and joint health, illustrating the complex interactions within microbiological ecosystems and their implications for medical science. The third most cited article, “Inflammaging and Osteoarthritis,” was published in Clinical Reviews in Allergy and Immunology (IF=11.3) in 2023 and has accumulated 193 citations [28].

### 3.2 Global Research Contributions and Collaborations

This bibliometric analysis highlighted significant contributions from 37 countries and regions, with the top 10 most productive nations accounting for 89.16% of the 584 articles analyzed. China emerged as the leader with 227 publications, followed by the United States with 162 and Canada with 57. This indicates a broad international interest spanning continents, including Asia, North America, Europe, and Australia (Table **1**). Although the United States ranks second in total publications, it leads in total citations with 2,075, which underscores the high impact of its research outputs. China, despite having the highest number of publications, closely follows with 2,044 citations, demonstrating its vital role in both volume and collaborative efforts.

Fig. (**3**) illustrates the international collaborations among 23 countries in the study of gut microbiota and OA, requiring a minimum of three articles for inclusion. This visualization highlights the active engagement of these countries, with China at the forefront, and showcases the most extensive collaborative ties, with a total link strength of 44. It is followed by the United States with a total link strength of 36, and the UK with a score of 23. The robust interconnections among countries are represented by nodes for individual nations and lines depicting their collaborative ties. China’s central position in the network is emphasized by a larger node size and diverse link colors, underscoring its significant role in driving international collaborations. This positioning not only highlights China’s pivotal contributions but also its influence in shaping global research directions in this field, reinforcing its status as a key player in the global research community focused on gut microbiota and OA. Furthermore, the map delineates seven distinct clusters of collaboration, suggesting regional and thematic affinities in research efforts. The United States, China, and the UK are prominently featured at the core of the top three major cooperation groups, indicating intense institutional cooperation.

### 3.3 Institutional Contributions and Collaborations

Fig. (**4A**) presents the top 10 institutions leading research on the relationship between gut microbiota and osteoarthritis. The University of Calgary stands out as the most prolific institution, with 47 articles, followed by Central South University with 37 articles. Assistance Publique-Hôpitaux de Paris (AP-HP) ranks third, contributing 21 articles. Notably, several major U.S. institutions also appear prominently among the top contributors, including Harvard University (18 articles), University of Oklahoma Health Sciences Center (18), University of Oklahoma System (18), University of North Carolina (17), University of North Carolina at Chapel Hill (16), and Harvard University Medical Affiliates (15). In addition, the French institution INSERM (Institut National de la Santé et de la Recherche Médicale) contributes 15 publications.

This distribution highlights substantial research contributions from North America and Europe, with particularly strong output from the United States and Canada, alongside significant contributions from China and France.

Among the 75 institutions engaged in international collaborations with at least three articles, the Central South University and the University of Nottingham stand out with a total link strength of 21, indicative of extensive international collaborations. Harvard Medical School follows closely with a link strength of 16. The institutions are organized into several distinct clusters, each representing varying levels of inter-institutional cooperation (Fig. **4B**). Among these, the University of Nottingham emerges as a central hub, connecting multiple major institutions, including Central South University, Harvard Medical School, Massachusetts General Hospital, and Fudan University, within the largest collaboration cluster. Other prominent clusters include the University of Calgary and its affiliated partners, as well as networks anchored by the University of Oklahoma and the University of Helsinki. Notably, strong collaborative ties are evident among institutions in China, the United States, and Europe, underscoring the international and multidisciplinary nature of research in this field. This collaborative landscape illustrates how leading institutions from North America, Europe, and Asia have established active research networks, fostering the global advance in understanding the gut microbiota–osteoarthritis relationship.

### 3.4 Analysis of the Authors

A total of 1,228 authors have contributed to publications in this field. As shown in Table **2**, the top 20 most productive authors include Hart David and Reimer Raylene, each with 9 publications since 2016, followed by Jeffries Matlock with 6 publications. Notably, Huang Zeyu has emerged as the most cited author, with 285 citations, closely followed by Hart David and Reimer Raylene, each cited 253 times. Fig. (**5**) visualizes the co-authorship network among various authors, revealing the collaborative dynamics within the research community. Among the 124 authors engaged in international collaborations with at least two articles, Lei Guanghua, Wei Jie, and Yang Tuo have the highest collaborative influence, each with a total link strength of 51. This figure also identifies two distinct clusters of collaboration, indicating close cooperation among these prolific authors.

### 3.5 Analysis of Journals

Table **3** presents the journals that have contributed to research on the association between gut microbiota and OA from 2008 to 2025. Among these, the top 10 journals had IF for 2023 ranging from 3 to 20.3 Notably, *Osteoarthritis and Cartilage* is the most influential journal, as evidenced by its highest H-index of 8. It also leads in total publication (8 articles) and citations, indicating significant recognition within the field.

The co-citation analysis, illustrated in Fig. (**6A**), shows connections between 31 journals based on how frequently they are cited together. This analysis highlights *Osteoarthritis and Cartilage*, *Arthritis and Rheumatology*, and *Annals of the Rheumatic Diseases* as the most interconnected journals, with total link strengths of 91, 36, and 34, respectively. These metrics demonstrate their pivotal roles in the research network. Fig. (**6B**) presents the journal coupling networks, indicating common research themes among 31 journals, each with at least five connections. *Osteoarthritis and Cartilage, Arthritis and Rheumatology*, and *Frontiers in Microbiology* stand out with the highest link strengths of 763, 565, and 488, respectively. These journals are central to the field, driving forward the discussion on the role of gut microbiota in OA through their publication volume, influence, and thematic relevance.

### 3.6 Keyword Analysis

A comprehensive keyword co-occurrence analysis was performed on 1,571 articles on gut microbiota and OA, yielding 529 distinct keywords. Using “Author Keywords” as the primary data source, seven major thematic clusters were identified in the co-occurrence network (Fig. **7A**). Each cluster reflects a distinct research focus in the field.

**Cluster 1 (Blue): Gut Microbiome and Systemic Associations**.

 This cluster centers on broader, systemic concepts, such as “gut microbiome,” “association,” “epidemiology,” “immunity,” “identification,” and “fluid,” highlighting studies that explore the link between gut microbiota and OA in the context of systemic health, diagnostics, and epidemiological trends.

**Cluster 2 (Green): Metabolic Syndrome and Experimental Models**:

 Key terms include “arthritis,” “hip,” “metabolic syndrome,” “diet-induced obesity,” “leptin,” and “mice,” indicating a focus on metabolic risk factors, animal models, and the mechanistic pathways by which metabolic disorders intersect with OA pathogenesis.

**Cluster 3 (Light Blue): Experimental Interventions and Methodology**:

 This cluster features keywords like “animal model,” “chondroitin sulfate,” “double-blind,” “glucosamine,” and “supplementation,” underscoring research into OA interventions, methodological rigor, and the use of animal models to test therapeutic strategies.

**Cluster 4 (Yellow-Green): Cartilage Biology and Disease Progression**:

 Keywords, such as “cartilage,” “macrophages,” “microbiome,” “progression,” “pathogenesis,” and “chondrocytes,” indicate an emphasis on tissue-level mechanisms, immune cell involvement, and the biological processes underlying OA progression.

**Cluster 5 (Red): Obesity, Inflammation, and Clinical Traits**:

 This cluster includes “obesity,” “inflammation,” “adipose tissue,” “knee osteoarthritis,” and “risk,” reflecting a focus on the clinical associations between metabolic inflammation, adiposity, and OA severity, particularly in the knee.

**Cluster 6 (Yellow): Disease Burden and Management**: Key terms, such as “burden,” “cells,” “degradation,” “management,” and “prevalence,” point to research on the clinical impact, management approaches, and the cellular mechanisms driving OA pathology.

**Cluster 7 (Purple): Molecular Markers and Disease Severity**:

 This small but distinct cluster is characterized by “expression,” “immune-system,” “murine model,” “resistance,” and “severity,” highlighting studies on molecular biomarkers and experimental disease models to evaluate OA severity and the immune response.

Within the overall network, the most prominent keywords by frequency and centrality are “gut microbiota” (61 occurrences, link strength 251), “inflammation” (54, 231), “knee osteoarthritis” (31, 133), and “osteoarthritis” (37, 122). These terms anchor the thematic landscape, reflecting the central role of gut-joint interactions, inflammatory processes, and specific OA phenotypes in current research. Other high-frequency terms, such as “obesity,” “cartilage,” “progression,” and “expression,” underscore the importance of metabolic risk factors, tissue degeneration, and molecular investigations in the field.

The temporal visualization (Fig. **7B**) demonstrates a gradual thematic evolution, with recent years (2021–2023) showing increased attention to terms related to advanced methodologies and clinical translation, such as “expression,” “double-blind,” “*in vitro*,” and “Mendelian randomization.” This suggests a growing emphasis on molecular mechanisms, translational research, and methodological rigor in the latest research outputs.

### 3.7 Analysis of Burst Keywords

Citation burst analysis (Fig. **8**) highlights the shifting research priorities over time by identifying keywords with the strongest citation bursts and their temporal patterns. The burst keywords, in order of their appearance and duration, are as follows: Early bursts (2012–2017/2020): “chondroitin sulfate” (2012–2020), “animal model” (2012–2019), and “osteoarthritis” (2012–2017) were among the earliest strong citation bursts, reflecting a foundational focus on therapeutic interventions, preclinical models, and disease characterization. Mid-phase (2016–2020): Keywords like “obesity”, “adipose tissue”, and “bacteria” (2016–2020/2017) began to gain prominence, indicating a growing interest in metabolic risk factors and microbiome composition in OA etiopathogenesis. Recent bursts (2017–2025): “Inflammation” (2017–2018) and “hip” (2019–2021) mark increased attention toward inflammatory mechanisms and specific joint involvement. From 2020 onward, there is a notable surge in methodological and translational terms: “expression” (2020–2021), “health” (2020–2021), “double blind” (2020–2021), and “*in vitro*” (2022–2023), reflecting an increasing focus on molecular markers, health outcomes, and experimental rigor. The most recent citation bursts are observed for “rheumatoid arthritis” (2022–2023), “mendelian randomization” (2023–2025), and “progression” (2023–2025), indicating emerging research frontiers in genetic epidemiology and longitudinal disease tracking.

## 4 DISCUSSION

This bibliometric analysis provides a systematic overview of the research landscape linking gut microbiota and OA over the past two decades. Although the sample size is moderate, it reflects the emerging, highly specialized nature of the field of gut-joint axis research, as evidenced by comparisons with similar published bibliometric studies [21, 22]. Our findings reveal a marked increase in research activity since 2015, with a particularly strong surge after 2020, reflecting the growing recognition of the gut-joint axis as a crucial area of investigation in OA pathogenesis and management.

The bibliometric results reveal the appearance of highly cited articles, such as “Metformin: Mechanisms in Human Obesity and Weight Loss,” which, while not exclusively focused on OA, was included due to its intersection with gut microbiota and metabolic regulation. Metformin, a first-line therapy for type 2 diabetes, exerts part of its therapeutic effect by modulating gut microbiota composition and function, thereby impacting systemic inflammation and metabolic homeostasis [27]. Recent studies have explored the use of metformin in OA models, demonstrating its potential to modulate the gut-joint axis through anti-inflammatory effects and microbiota-mediated pathways [29]. Thus, the inclusion of metformin-related literature is justified, as it reflects the evolving interest in metabolic interventions that may intersect with OA management through the gut microbiome [5, 8, 10]. However, this also underscores the importance of refining bibliometric search strategies to balance comprehensiveness with specificity.

Our analysis demonstrated that China, the United States, and Canada are the most productive countries in this domain. China’s centrality is attributable not only to the sheer volume of publications but also to its extensive international collaborations, particularly with North America and Europe. Previous bibliometric studies corroborated China’s leading role in biomedical research, often facilitated by co-authorship networks and joint projects with high-impact institutions [22]. For example, collaborative ties between Central South University and institutions in the US and Europe have significantly contributed to the advancement of gut microbiota–OA research, reinforcing China’s influence through both productivity and global partnership [22, 26].

Thematic clustering of keywords reveals several major research directions:

**Cluster 1: Gut Microbiome and Systemic Associations**

Research in this cluster has shifted towards understanding the systemic impact of the gut-joint axis, with large-scale epidemiological studies exploring the influence of gut dysbiosis on OA risk and comorbidities [6, 7]. These findings suggest that large-scale cohort studies and multi-omics analyses are warranted to clarify causal relationships and to identify predictive microbial biomarkers for early OA detection and risk stratification [8, 9]. Moreover, the “axis” concept extends beyond the gut-joint interaction, with emerging evidence linking gut microbiome perturbations to extra-articular manifestations, such as cardiovascular and metabolic comorbidities in OA patients [9, 10]. This systemic perspective is crucial for the development of integrated prevention and intervention strategies.

**Cluster 2: Metabolic Syndrome and Experimental Models**

The second cluster focuses on metabolic risk factors, such as “obesity,” “metabolic syndrome,” “diet-induced obesity,” and “leptin,” and their experimental modeling in OA. Metabolic syndrome has long been recognized as a significant risk factor for OA progression, independent of mechanical loading [1, 4]. Preclinical models, including high-fat diet-induced obesity in mice, have provided compelling evidence that gut microbiota alterations mediate the pro-inflammatory state and cartilage degradation associated with metabolic OA [5, 30]. For example, Schott *et al*. demonstrated that a high-fat di*et al*ters gut microbial composition and increases systemic endotoxin levels, which in turn exacerbate joint inflammation and cartilage damage in mouse models [5]. The integration of metabolic and immunological pathways in animal studies has enhanced our understanding of how metabolic dysfunction translates into joint pathology, underscoring the translational potential of targeting the gut microbiota-metabolism axis for OA therapy [10, 12]. However, it is crucial to acknowledge limitations in model systems and to validate experimental findings in well-designed human studies.

**Cluster 3: Experimental Interventions and Methodology**

Keywords in this cluster, including “animal model,” “chondroitin sulfate,” “glucosamine,” “double-blind,” and “supplementation,” reflect the increasing rigor and diversity of experimental approaches in OA research. The field has shifted from descriptive studies to intervention trials aimed at modulating the gut microbiome or testing nutraceuticals and supplements for OA prevention and treatment [17, 20]. Notably, the inclusion of “double-blind” as a frequent and burst keyword signals the growing adoption of high-quality randomized controlled trials (RCTs), which are essential for generating robust clinical evidence [31]. Nutritional interventions, such as prebiotic/probiotic supplementation and dietary modifications (*e.g*., the Mediterranean diet), have shown promise in modifying gut microbiota and reducing inflammatory markers in OA patients [17, 19]. However, there remains considerable debate regarding the efficacy of widely used supplements like chondroitin sulfate and glucosamine, with meta-analyses revealing variable outcomes depending on study design and participant characteristics [20]. The cluster thus illustrates the methodological advancements and ongoing controversies in translating microbiome science into clinical benefit.

**Cluster 4: Cartilage Biology and Disease Progression**

This cluster highlights the intricate interplay between cartilage biology, immune cell dynamics, and OA progression. Keywords, such as “cartilage,” “chondrocytes,” “macrophages,” “progression,” and “pathogenesis,” reflect research that investigates how gut-derived factors influence joint tissues at the molecular and cellular level. There is substantial evidence that gut microbiota-derived metabolites, such as short-chain fatty acids (SCFAs), modulate immune responses and tissue homeostasis in OA [10, 11]. Specifically, butyrate has been shown to enhance regulatory T cell (Treg) function and suppress pro-inflammatory cytokine production, thereby exerting chondroprotective effects [11, 32]. Conversely, dysbiosis-driven increases in lipopolysaccharide (LPS) levels activate TLR4/NF-κB signaling in synovial macrophages, fueling a cycle of inflammation and cartilage breakdown [30, 31, 33]. These mechanisms are further supported by animal studies showing that fecal microbiota transplantation from OA donors accelerates joint degeneration in recipient mice [34]. Collectively, these findings support the therapeutic targeting of gut-joint immunometabolic pathways to slow or halt OA progression.

**Cluster 5: Obesity, Inflammation, and Clinical Traits**

The fifth cluster centers on “obesity,” “inflammation,” “adipose tissue,” “knee osteoarthritis,” and “risk,” highlighting the clinical significance of metabolic inflammation in OA pathophysiology. Obesity is now understood to promote OA not only through increased mechanical load but also through the secretion of pro-inflammatory adipokines and alterations in gut microbiota composition [1, 6, 8]. Elevated levels of systemic inflammatory mediators, including TNF-α, IL-1β, and IL-6, have been consistently linked to both metabolic and cartilage degeneration phenotypes in OA [4, 10]. The gut microbiota acts as a mediator of this low-grade inflammation. Studies demonstrated that obese individuals exhibit gut dysbiosis, enhanced intestinal permeability, and increased circulating LPS, all contributing to joint pathology [5, 35, 36]. These insights reinforce the importance of integrating weight management, dietary modification, and microbiota-targeted interventions in the comprehensive management of OA.

**Cluster 6: Disease Burden and Management**

Cluster 6 encompasses keywords related to the clinical burden of OA, such as “burden,” “management,” “prevalence,” and “validation.” This reflects a growing emphasis on population-level impact, healthcare resource allocation, and the validation of new diagnostic and therapeutic approaches [37, 38]. Epidemiological research has shown a steady increase in OA incidence and prevalence, paralleling worldwide trends in obesity, aging, and metabolic disease [37, 38]. The economic and social burden of OA underscores the urgency of developing cost-effective, evidence-based management strategies [39]. Recent advances in microbiome science offer potential for novel diagnostics, predictive biomarkers, and personalized interventions, but require rigorous validation in diverse populations and healthcare settings [22]. This cluster thus points to a translational research agenda that bridges fundamental discovery with clinical implementation and health policy.

**Cluster 7: Molecular Markers and Disease Severity**

The final cluster, characterized by “expression,” “immune-system,” “murine model,” “resistance,” and “severity,” marks the forefront of molecular OA research. Investigations into gene and protein expression profiles in joint tissues and circulating immune cells aim to unravel disease heterogeneity and identify molecular signatures associated with OA severity and therapeutic response [10, 11, 14]. Recent studies employing multi-omics and systems biology approaches have begun to dissect the complex interplay between host genetics, microbial factors, and immune regulation [13, 14]. For example, transcriptomic analyses have linked altered expression of immune-related genes to specific gut microbial patterns in OA models [13]. Moreover, research into immune resistance mechanisms and inflammatory cell infiltration provides mechanistic insights into why some patients experience rapid disease progression while others remain clinically stable [14, 40]. These advances are paving the way for precision medicine approaches that tailor therapy according to molecular and microbial profiles.

Keyword burst analysis provides insights into the evolving research landscape. Between 2015 and 2018, frequently cited terms included “gut microbiota,” “inflammation,” and “osteoarthritis,” reflecting the early-stage exploration of the gut-joint axis [39, 41]. During this period, foundational studies established the link between gut dysbiosis, systemic inflammation, and joint degeneration, laying the groundwork for mechanistic research. From 2018 to 2021, research shifted toward molecular and immunological mechanisms, with keywords, such as “IL-6,” “TLR4,” “Wnt signaling,” and “SCFAs” gaining prominence [42]. This shift indicates a growing interest in understanding the molecular pathways through which gut microbiota influences OA. Additionally, the emergence of “double-blind” and “clinical trial” around 2020 highlights an increasing emphasis on evidence-based methodologies [43], signaling a shift from observational studies to interventional trials assessing microbiome-targeted therapies [31]. Looking forward, the field is poised to benefit from advances in multi-omics, precision probiotics, and exosome-based communication between gut microbes and joint tissues [44]. Collaborative, multidisciplinary research will be key to translating bibliometric and mechanistic insights into clinical innovation [45-47].

## 5 LIMITATION

Despite the insights provided by this analysis, several limitations should be acknowledged. First, our study relied exclusively on the WoSCC as the data source. While WoSCC is widely used in bibliometric studies and offers rigorous indexing standards, its coverage may differ from that of other major databases, such as Scopus, potentially leading to discrepancies in the number and scope of included articles [48]. Recent research has reported that Scopus often contains a broader range of healthcare-related publications and may yield different results for the same journal or topic. Thus, our findings may not capture all relevant literature, especially studies indexed only in Scopus or other databases. Second, restricting publications to English-language publications introduces a language bias, potentially underrepresenting important contributions from non-English-speaking regions, which could affect the global perspective on the research landscape [49]. Third, bibliometric analyses are inherently subject to citation-related biases, including time-lag bias (where recent influential articles may not have accumulated sufficient citations), self-citation effects, and the variable influence of different article types. Although we excluded review articles to enhance topic specificity, highly cited articles that are only tangentially related to the core topic may still appear due to the nature of citation networks. Fourth, the use of citation counts as a proxy for research impact does not necessarily reflect clinical significance or translational value, and may be influenced by factors, such as journal prestige, open-access status, and self-citation practices. Fifth, our analysis utilized multiple bibliometric tools (VOSviewer, CiteSpace, and bibliometric.com), each with its own technical limitations, visualization algorithms, and parameter settings. Differences in data handling, clustering methods, and visualization outputs between these tools may influence the interpretation of collaboration networks or thematic structures. Finally, the dynamic nature of bibliographic databases means that results may vary depending on the exact time of data retrieval, and the rapidly evolving field may soon render some findings outdated.

Future bibliometric studies should consider integrating multiple databases, broadening language inclusion, and employing advanced methods to correct for citation-related biases and tool-specific limitations. Combining quantitative mapping with qualitative or systematic reviews may further enhance the translational relevance and comprehensiveness of research in this area.

## CONCLUSION

This bibliometric analysis highlights the evolving research landscape linking gut microbiota to OA. It identifies “gut microbiota,” “inflammation,” and “obesity” as key themes influencing research and therapeutic advancements. While the analysis reveals significant global contributions, especially from China, the United States, and Canada, its reliance on citation metrics and the exclusion of non-English studies emphasizes the need for more inclusive research methods to gain a comprehensive understanding of the gut-joint axis.

## Figures and Tables

**Fig. (1):**
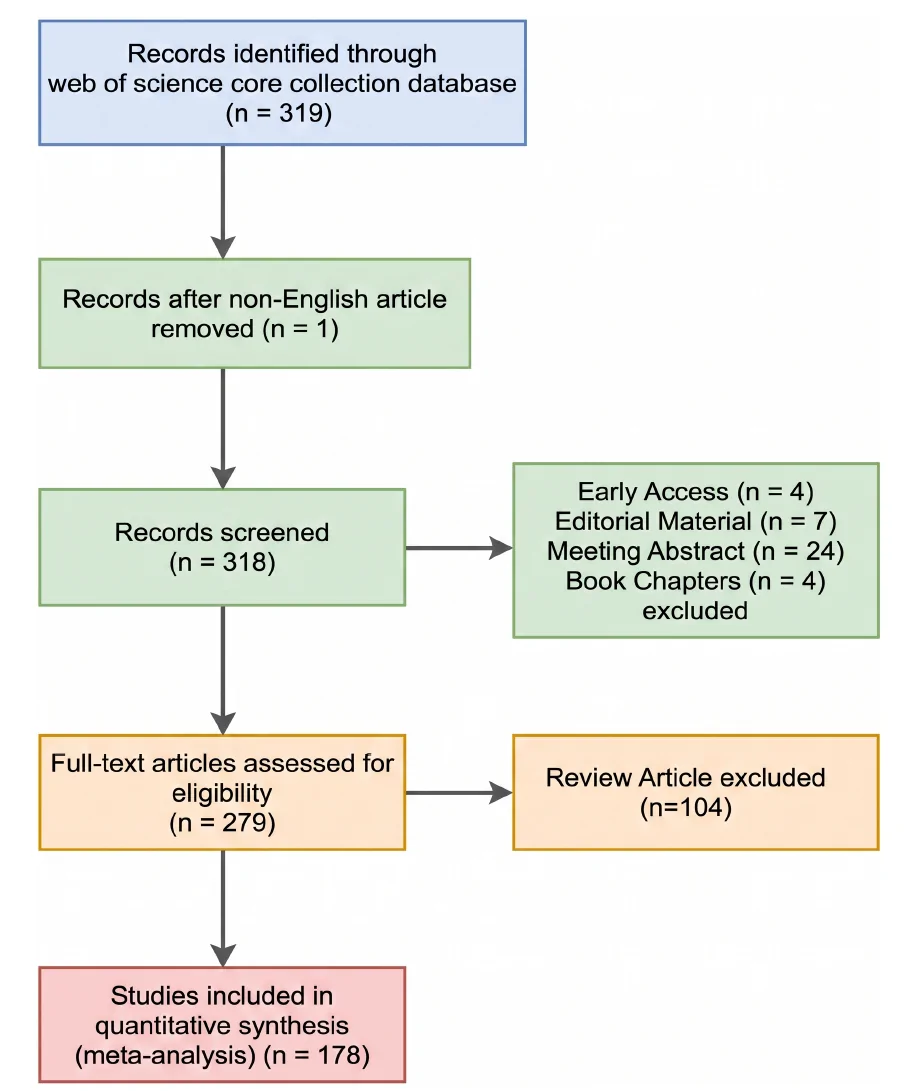
Flowchart depicting the literature screening process for studies on the association between gut microbiota and osteoarthritis.

**Fig. (2):**
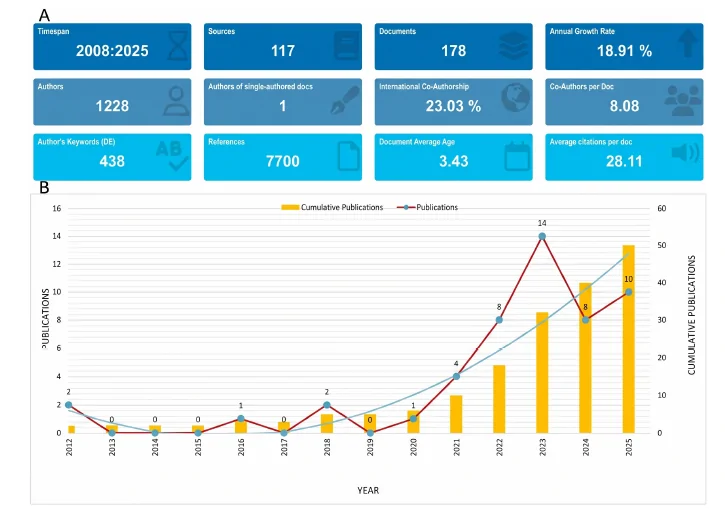
Publication trends and citation impact in gut microbiota and osteoarthritis research. **(A)** Overview of the publications on gut microbiota and osteoarthritis. (**B)** Annual publication trends in apoptosis in gut microbiota and osteoarthritis from 2002 to 2024.

**Fig. (3):**
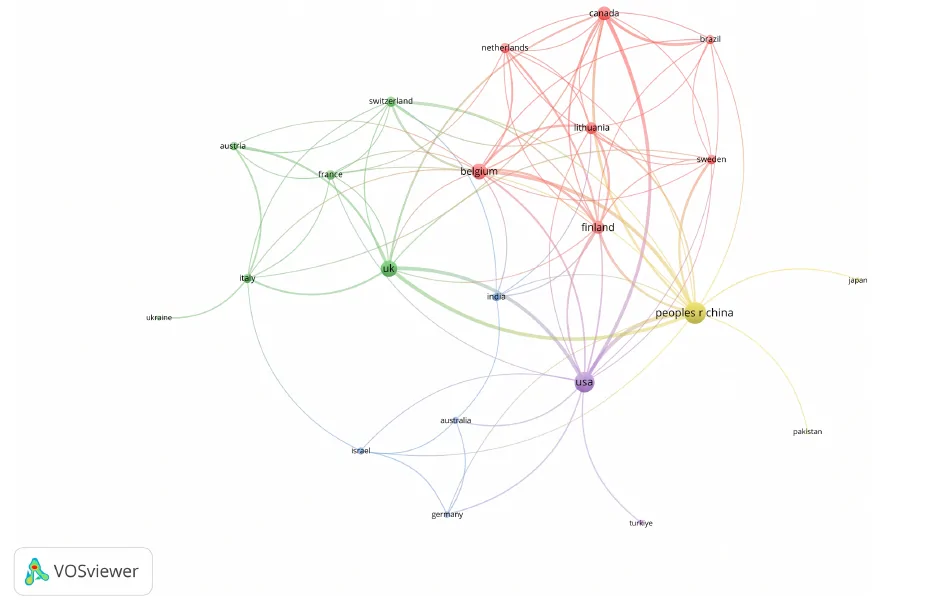
Collaboration in gut microbiota and osteoarthritis research.

**Fig. (4):**
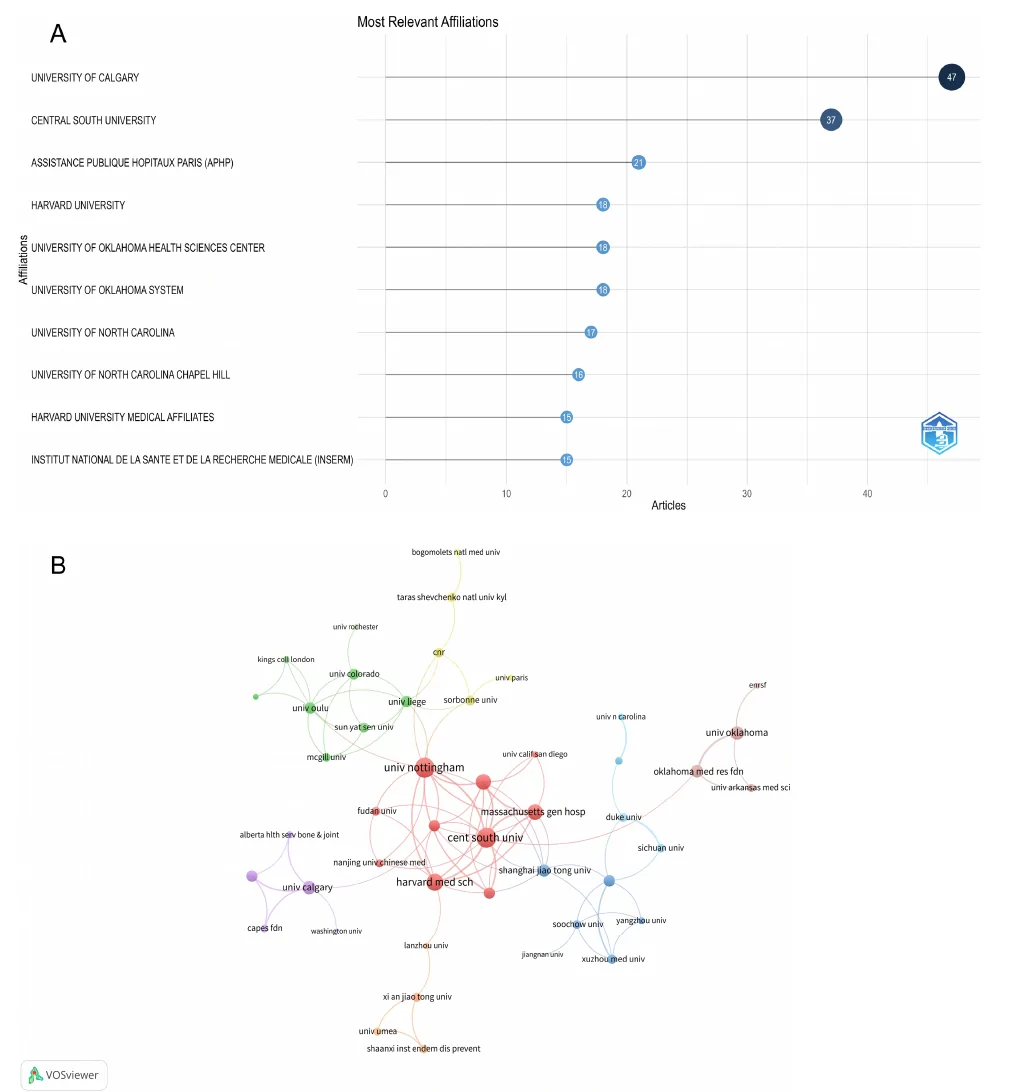
Institutional Contributions and Collaborations in Gut Microbiota and Osteoarthritis Research. (**A**) Top 10 institutions spearheading research on the association between gut microbiota and OA. (**B**) Author collaboration network in apoptosis in gut microbiota and osteoarthritis. Node size indicates publication volume, and link thickness represents the strength of co-authorships. Different colors signify distinct research clusters, and total link strength quantifies the extent of collaborative interactions among authors.

**Fig. (5):**
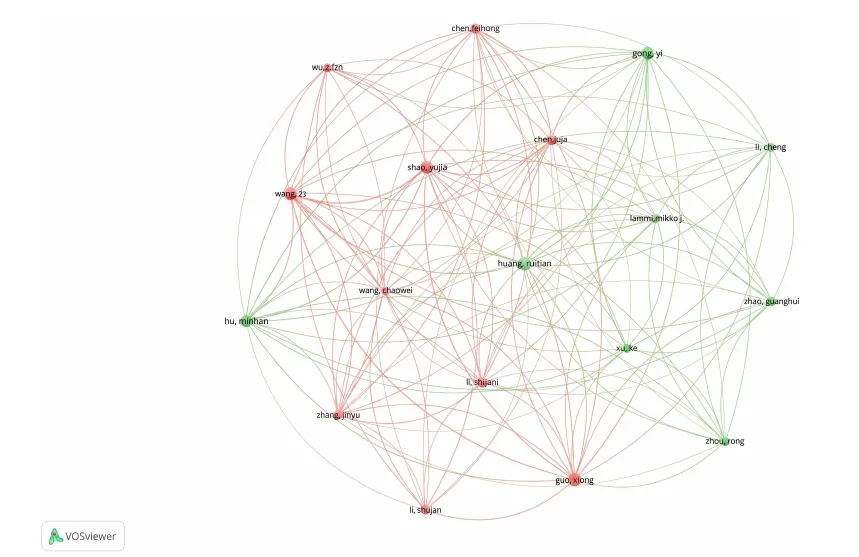
Author contributions and collaborations in gut microbiota and osteoarthritis research.

**Fig. (6):**
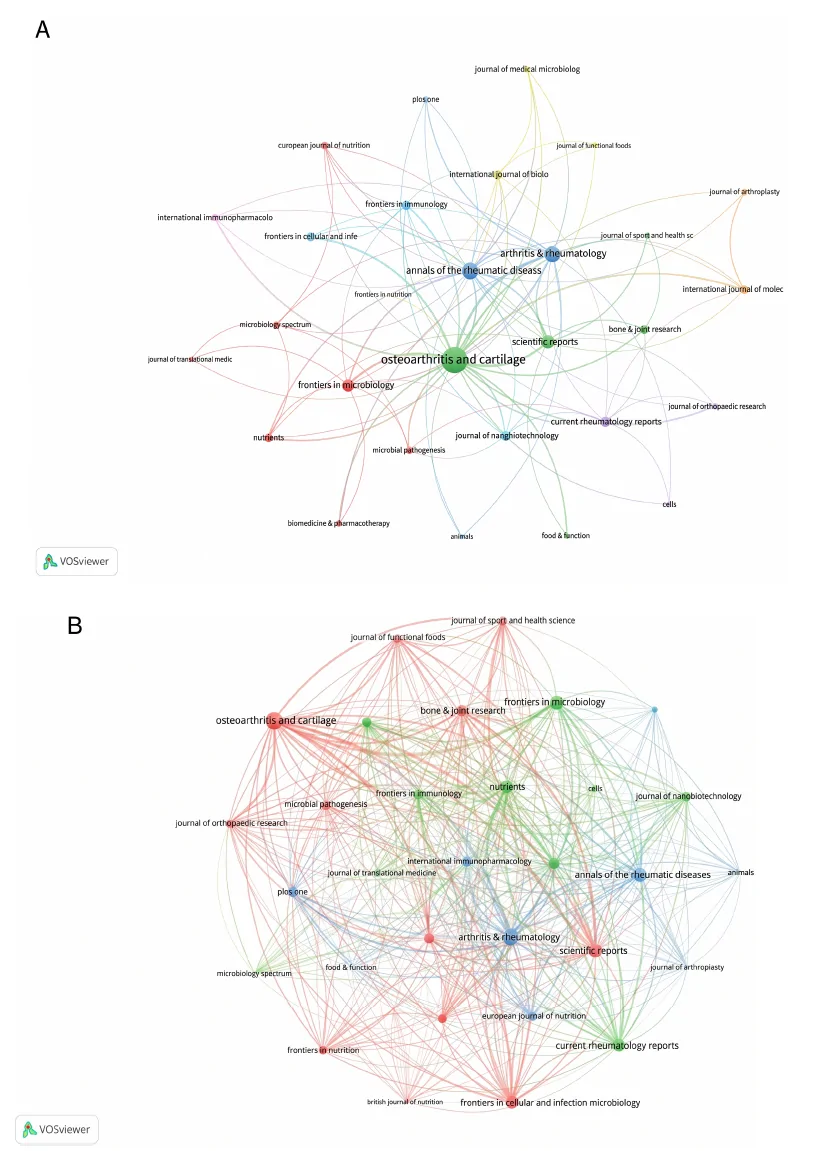
Journal Network Analysis in Gut Microbiota and Osteoarthritis Research. **(A).** Co-occurrence network depicting thematic connections between journals, illustrating citation patterns within articles. **(B).** Coupling network illustrating intellectual linkages between journals through shared references, emphasizing commonly cited works.

**Fig. (7):**
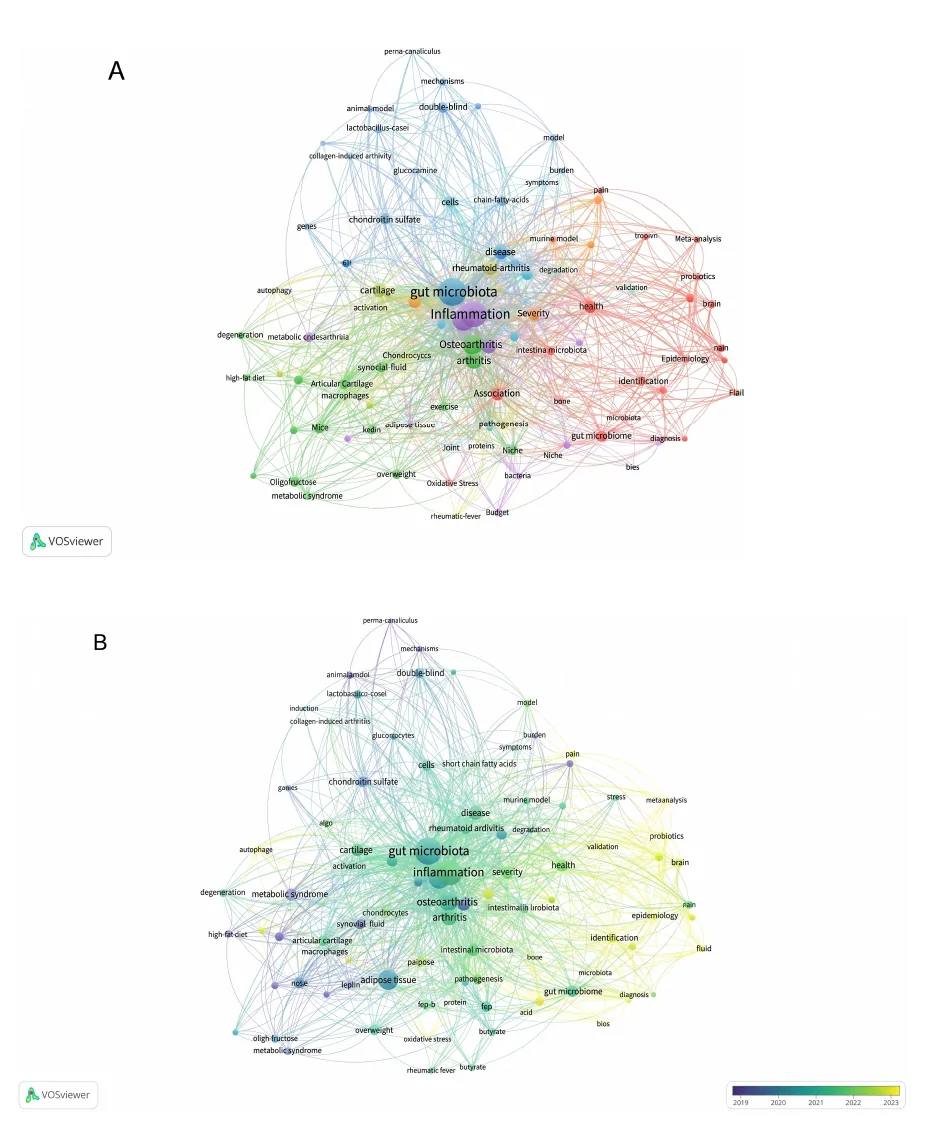
Keyword Co-occurrence Network in Gut Microbiota and Osteoarthritis Research. (**A**). Visual analysis of the keyword co-occurrence network by different clusters. (**B**). Visual analysis of the keyword co-occurrence network in accordance with the appearance of the average time. Nodes represent keywords, sized by their frequency of occurrence. Links indicate co-occurrences, with line thickness reflecting the strength of the association. Colors represent the average publication year of articles associated with each keyword, as shown by the color gradient.

**Fig. (8):**
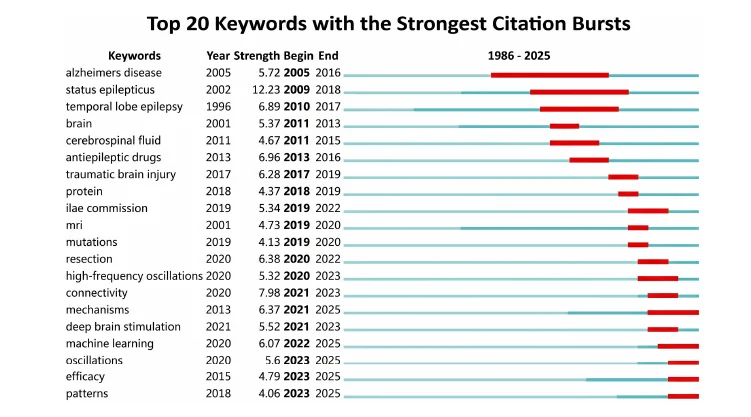
Citation Burst Analysis for Keywords in Apoptosis in Gut Microbiota and Osteoarthritis Research. Chart of the top 10 keywords with significant citation bursts between 2004 and 2024, with blue lines marking the analysis period and red lines highlighting the burst phases.

**Table 1:** Publication and citation profiles of leading countries.

**Country**	**Documents**	**Citations**	**Total Link Strength**
**China**	89	2044	44
**USA**	50	2075	36
**UK**	11	288	23
**Belgium**	5	175	20
**Canada**	19	616	18
**Finland**	3	44	16
**Lithuania**	2	38	13
**Italy**	11	483	11
**Sweden**	3	56	11
**Brazil**	3	126	10
**Netherlands**	3	251	10
**Switzerland**	2	141	10
**France**	5	153	8
**Austria**	2	169	7
**India**	2	44	6
**Israel**	3	80	5
**Australia**	9	226	4
**Germany**	2	2	4
**Japan**	2	35	2
**Ukraine**	5	109	2
**Pakistan**	2	12	1
**Turky**	3	0	1
**South Korea**	5	155	0

**Table 2:** Publication and citation profiles of high-impact authors.

Author	h_index	g-index	m-index	PY_start	TP	TP_Frac	TP_rank	TC	TC_rank
Hart David A.	7	9	0.70	2016	9	1.42	1	253	2
Reimer Raylene A.	7	9	0.70	2016	9	1.42	2	253	2
Jeffries Matlock A.	5	6	0.83	2020	6	0.73	4	99	15
Dunn Christopher M.	4	4	0.67	2020	4	0.26	11	88	19
Doherty Michael	3	4	0.60	2021	4	0.35	10	88	19
Gong Yi	3	3	0.60	2021	3	0.37	21	48	34
Hernandez Christopher J.	3	4	0.33	2017	4	0.29	13	54	28
Hu Minhan	3	3	0.60	2021	3	0.47	23	48	34
Huang Ruitian	3	3	0.60	2021	3	0.22	24	48	34
Huang Zeyu	3	3	0.30	2016	3	0.55	25	285	1
Korotkyi Oleksandr	3	3	0.43	2019	3	0.22	27	73	25
Lei Guanghua	3	5	0.60	2021	5	0.35	6	155	7
Li Jiatian	3	4	0.75	2022	4	NA	NA	92	16
Park Sung-Hwan	3	4	0.60	2021	4	0.17	31	49	30
Prinz Emmaline	3	3	0.40	2019	3	0.40	32	81	24
Rios Jaqueline L.	3	3	1.50	2024	3	0.55	33	26	42
Schlupp Leoni	3	3	0.30	2016	3	0.30	34	118	13
Velasco Cassandra	3	3	1.50	2024	3	0.38	36	26	42
Vitetta Luis	3	3	0.50	2020	3	0.37	37	84	23
Wang Xi	3	3	0.23	2013	3	0.26	16	87	22

**Table 3:** Bibliometric indicators of high-impact journals.

**Journal**	**H_index**	**G_index**	**M_index**	**IF 2023**	**JCR 2023**	**TP**	**TP_rank**	**TC**	**TC_rank**	**PY_start**
Osteoarthritis and cartilage	8	8	0.727	7.2	Q1	8	1	602	1	2015
Arthritis & rheumatology	5	6	0.833	11.4	Q1	6	2	159	6	2020
Nutrients	5	6	1.000	4.8	Q1	6	3	151	7	2021
Scientific reports	4	6	0.400	3.8	Q1	6	4	160	5	2016
Annals of the rheumatic diseases	3	3	0.500	20.3	Q1	3	8	206	2	2020
Current rheumatology reports	3	3	0.333	5.7	Q1	3	9	27	71	2017
Food & function	3	3	0.429	5.1	Q1	3	10	37	40	2019
Frontiers in cellular and infection microbiology	3	4	0.250	4.6	Q1	4	6	43	31	2014
Frontiers in immunology	3	3	0.333	5.7	Q1	3	11	94	14	2017
Frontiers in microbiology	3	5	0.333	4	Q2	5	5	94	15	2017
International journal of biological macromolecules	3	4	0.300	7.7	Q1	4	7	24	80	2016
Biomedicine & pharmacotherapy	2	2	0.667	6.9	Q1	2	14	35	44	2023
Bone & joint research	2	2	0.250	4.7	Q1	2	15	14	129	2018
British journal of nutrition	2	2	0.111	3	Q2	2	16	31	57	2008
Cells	2	2	0.500	5.1	Q2	2	17	32	52	2022
European journal of nutrition	2	2	1.000	4.1	Q2	2	18	18	103	2024
Frontiers in nutrition	2	3	0.333	4	Q2	3	12	22	87	2020
International immunopharmacology	2	2	0.667	4.8	Q1	2	20	16	115	2023
International journal of molecular sciences	2	2	0.333	4.9	Q2	2	21	99	13	2020
Journal of functional foods	2	2	0.333	3.8	Q2	2	23	14	135	2020

**Note(s):** H_index: The h-index of the journal, which measures both the productivity and citation impact of the publications. IF: Impact Factor, indicating the average number of citations to recent articles published in the journal. JCR_Quartile: The quartile ranking of the journal in the Journal Citation Reports, indicating the journal's ranking relative to others in the same field (Q1: top 25%, Q2: 25%-50%, Q3: 50%-75%, Q4: bottom 25%). TP: Total Publications. TP_rank: Rank of Total Publications. TC: Total Citations. TC_rank: Rank of Total Citations. Average Citations: The average number of citations per publication. PY_start: Publication Year Start, indicating the year the journal started publication.

## Data Availability

All data generated or analysed during this study are included in this published article and its Supplementary Information files.

## LIST OF ABBREVIATIONS

OA: Osteoarthritis
WHO: World Health Organization
LPS: Lipopolysaccharides
SCFAs: Short-Chain Fatty Acids
TLR4: Toll-Like Receptor 4
IL-6: Interleukin-6
IL-1β: Interleukin-1β
TNF-α: Tumor Necrosis Factor-Alpha
FMT: Fecal Microbiota Transplantation
WoSCC: Web of Science Core Collection
APHP: Assistance Publique–Hôpitaux de Paris
